# Factors associated with reported snoring among elderly patients attending the geriatric centre in Nigeria

**DOI:** 10.11604/pamj.2014.19.309.5244

**Published:** 2014-11-21

**Authors:** Lawrence Adekunle Adebusoye, Adetola Morenikeji Ogunbode, Olufemi Oluwole Olowookere

**Affiliations:** 1 Department of Family Medicine, University College Hospital, Ibadan, Nigeria

**Keywords:** Snoring, elderly, geriatric clinic, Nigeria

## Abstract

**Introduction:**

Snoring is the major symptom of sleep disordered breathing (SDB) which is of immense public health importance. It is associated with some morbidities and mortality in the elderly. Few studies have addressed this problem in the elderly Nigerians.

**Methods:**

Cross-sectional study of 843 elderly patients at the Geriatric Centre, University College Hospital, Ibadan, Nigeria. Data were collected on the following candidate variables which may be associated with snoring such as socio-demographic characteristics, morbidities, lifestyle habits and functional disability using Katz index. Anthropometric measurements such as body mass index and neck, waist and hip circumferences were taken. Statistical analysis was done with SPSS 17.

**Results:**

The point prevalence of reported snoring was 31.2%. Habitual snoring was reported by 24.8%. Snoring was significantly associated with obesity, moderate to severe oropharyngeal crowding, wide neck and waist circumferences in both sexes. Logistic regression analysis showed wide neck circumference (OR = 6.005; 95%CI= 2.150-16.770) among the males and obesity (OR = 2.028; 95%CI= 1.344-3.061) and moderate to severe oropharyngeal crowding (OR = 1.639; 95%CI= 1.057-2.543) in the females to be the most significant factors associated with snoring.

**Conclusion:**

The high prevalence of snoring among elderly patients in Nigeria calls for concerted effort by healthcare workers to educate the elderly.

## Introduction

Snoring during sleep is caused by the vibration of soft tissue in the upper airways involving anatomical structures such as the soft palate, uvula, and the pharynx due to partial obstruction from narrowing that occurs in these structures during sleep [1, 2]. Historically in some cultures, snoring was seen as a symbol of a great man and large snoring sound was regarded as the evidence of a sound sleep [3]. However, snoring is associated with disturbance of partners, friends and family. Often, the snorer is usually unaware of his/her snoring [1].

The prevalence of snoring varies widely due to cultural, racial and study design used in obtaining the information. Similarly, it varies with age and sex of the population. Globally, the prevalence of snoring ranges between 16 to 89% (males 24 - 50% and females 14 – 30%) [4]. Among the elderly (aged 60 years and above), the prevalence of snoring was 39.4% with a male to female ratio of 1.64 [4]. Factors associated with snoring include male sex, increasing age, family history of snoring, obesity, wide neck circumference and tobacco smoking [5]. Similarly in Nigeria, male sex, old age, obesity, marital status and cigarette smoking were the factors reported in studies on snoring [1, 6].

Few studies had been carried out on snoring in Nigeria and they mostly centred on children and young adults. With the current shift of attention towards geriatric medicine in Nigeria, it is pertinent to have a database of medical illnesses experienced by this rapidly emerging group in order to enhance the knowledge of health workers and formulate policies towards their care. This study looked at the magnitude and factors associated with reported snoring among elderly patients at the first geriatric centre in Nigeria.

## Methods

**Study site:** this study was carried out at the outpatient unit of the Chief Tony Anenih Geriatric Centre (CTAGC), University College Hospital (UCH), Ibadan, south-western Nigeria with a population of 3.6 million people [7]. CTAGC is the first geriatric centre in Nigeria purposefully built for the care of elderly people on 17th November, 2012. The centre has speciality units such as physiotherapy, dietetics, geriatric lifestyle, ophthalmology, geriatric dentistry, memory and geriatric psychiatry units.

**Study design:** cross-sectional design was used for this study.

**Study population:** all elderly (60 years and above) patients who presented during the period of the study (January 15^th^ to April 30^th^, 2013) were recruited after obtaining their consent. Those who were too ill to participate in the study and those who did not consent were excluded. This study was part of a larger study on chronic sleep problems among the elderly.

**Sampling technique:** respondents were selected consecutively.

**Procedure:** the respondents were interviewed with a semi-structured questionnaire which was pre-tested before use. The questionnaire was administered in the presence of spouse, relations or bed mates as applicable who could corroborate the presence of snoring. Detailed information on their demographic characteristics, socio-economic and lifestyle habits were obtained by trained assistants. Comprehensive physical examination including anthropometric measurements was carried out by trained medical officers. The questionnaire was administered in English language and Yoruba language (the local dialect of most respondents) as necessary. The questionnaire took about 40 minutes to be administered.

### Basic activities of daily living (BADL)

This was assessed using Katz index of Independence in Activities of Daily Living. The Katz Index is the most appropriate instrument to assess functional status as a measurement of the patient's ability to perform activities of daily living independently [8]. Clinicians typically use the tool to detect problems in performing activities of daily living and to plan care accordingly [8]. The Index ranks adequacy of performance in the six functions of bathing, dressing, toileting, transferring, continence, and feeding. Respondents were scored yes/no for independence in each of the six functions. A score of 6 indicates full function, while 4 indicates moderate impairment, and 2 or less indicates severe functional impairment [8].

**Anthropometric measurements:** the stadiometre which was positioned on a flat surface was used to measure the height of the respondents to the nearest centimetre. The respondents were asked to remove their shoes, and their heels were positioned against the stand with their scapula, buttocks and heels resting against the wall. The weight of the respondents was measured with a weighing scale which was placed on a flat horizontal surface after they were asked to remove their personal effects. Their weights were measured to the nearest 0.1kg. At the end of each reading, the zero mark was checked for accuracy. The BMI of the patients was calculated by dividing weight (kilogrammes) by height in meters squared and this was graded using the WHO anthropometric classification [9]. Underweight was defined as BMI ^2^ and 18.5 – 24.9 kg/m^2^ as normal. Overweight was BMI 25.0 – 29.9kg/m^2^ and Obesity as BMI =30.0 kg/m^2^ [9].

### Waist-Hip Ratio (WHR) and Neck circumference

A flexible non-elastic measuring tape was used to measure the waist, hip and neck circumferences to the nearest 0.1cm. The hip circumference was measured at a level parallel to the floor, at the largest circumference of the buttocks. The waist circumference was measured at the end of several consecutive natural breaths, at a level parallel to the floor, midpoint between the top of the iliac crest and the lower margin of the last palpable rib in the mid axillary line. The waist circumference was used to identify individuals with increased risks for metabolic complications based upon threshold values of 80cm or greater for women and 94cm or greater for men as defined by the World Health Organization (WHO) and International Diabetic Federation (IDF) [10]. Waist to Hip ratio (WHR) was estimated by dividing waist circumference by hip circumference. The WHR threshold used for elderly women was 0.85 or more and 1.00 or more for men [10, 11]. Wide neck circumferences were taken as greater than 40cm in women and 43cm in men. This correlates strongly with the development of obstructive sleep apnoea [12].

**Throat examination:** the Mallampati visual assessment classification was used to assess oropharyngeal crowding during throat examination [13]. This was classified as follows; Class I: tonsils, pillars and soft palate were clearly visible; Class II: the uvula, pillars and upper pole were visible; Class III: only part of the soft palate was visible; the tonsils, pillars and base of the uvula could not be seen and Class IV: only the hard palate was visible [13].

### Ethical consideration

**Consent for the study:** ethical approval was received from the University of Ibadan/ UCH Institutional Ethical Review Board (NHREC/05/01/2008a). Informed consent of each respondent was obtained before examination and administration of questionnaire.

**Respondent's follow-up:** all the elderly patients recruited were treated for their primary complaints and those needing further evaluation were referred to specialist units within the hospital facility for further management of their conditions.

**Data analysis:** at the end of each day of the study, the administered questionnaires were sorted out, cross-checked after each interview and coded serially. Data entering, cleaning and analysis were carried out using SSPS (version 17). Descriptive statistics was used to describe socio-demographic characteristics of the respondents. Appropriate charts were used to illustrate categorical variables. Chi-square statistics was used to assess association between categorical variables. The values of significance were set at p < 0.05. Logistic regression analysis was used to explore relationship between significant variables and snoring.

## Results

**Figure 1:**
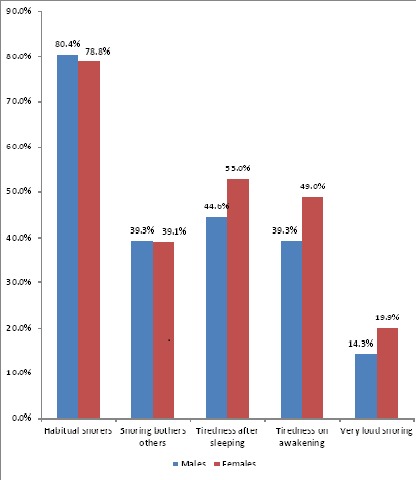
Sleep disorders reported by snorers by sex

Eight hundred and forty three respondents were included with a male to female ratio of 1 to 1.48. Their mean (SD) age was 69.3 (7.1) with a range (60 – 98) years. The majority of the respondents (76.5%) were in the young-old group (Table 1. Sleeping disorders experienced by the respondents who snored is shown in Figure 1. Of the 263 respondents who reported snoring, 79.5% of them (males 80.5% vs females 78.8%) were habitual snorers. Out of the number of respondents (263) who reported snoring, 39.2% reported that their snoring bothers others and 17.5% said their snoring was very loud. Similarly, 49.4% and 44.9% reported tiredness after sleeping and on awakening respectively.

**Table 1:** Socio-demographic characteristics by prevalence of snoring

	Snoring	
	YES = 263 n (%)	NO = 580 n (%)	TOTAL = 843 N (%)
**Age**			
60 - 75 years	208 (32.2)	437 (67.8)	645 (100.0)
75 – 84 years	48 (28.6)	120 (71.4)	168 (100.0)
≥85 years	7 (23.3)	23 (76.7)	30 (100.0)
**χ** ^**2**^ **= 1.736 df = 2; p = 0.420**			
**Sex**			
Male	112 (32.9)	228 (67.1)	340 (100.0)
Female	151 (30.0)	352 (70.0)	503 (100.0)
**χ** ^**2**^ **= 0.807 df = 1; p = 0.369**			
**Marital status**			
Currently married	173 (33.6)	342 (66.4)	515 (100.0)
Not currently married	90 (27.4)	238 (72.6)	328 (100.0)
**χ** ^**2**^ **= 3.535 df = 1; p = 0.060**			
**Educational status**			
No formal education	94 (28.6)	235 (71.4)	329 (100.0)
Primary	50 (27.9)	129 (72.1)	179 (100.0)
Secondary	52 (32.5)	108 (67.5)	160 (100.0)
Tertiary	67 (38.3)	108 (61.7)	175 (100.0)
**χ** ^**2**^ **= 6.186 df = 3; p = 0.104**			
**Occupational status**			
Still engaged in occupational activities	79 (32.6)	163 (67.4)	242 (100.0)
Not engaged in occupational activities	184 (30.6)	417 (69.4)	601 (100.0)
**χ** ^**2**^ **= 0.331 df = 1; p = 0.565**			
**Financial support**			
Depends on others	202 (31.3)	443 (68.7)	645 (100.0)
Self-supporting	61 (30.8)	137 (69.2)	198 (100.0)
**χ** ^**2**^ **= 0.018 df = 1; p = 0.892**			

Table 2 shows the basic activities of daily living by the prevalence of reported snoring. Overall, a few of the respondents 43 (5.3%) had some degree of functional disability in carrying out their activities of daily living with 34.9% reporting snoring. There was no significant association between basic activities of daily living such as bathing, toileting, dressing, transferring, continence, feeding and the prevalence of reported snoring.

**Table 2:** Basic Activities of Daily Living (BADL) by prevalence of snoring

	SNORING		
Basic Activities of Daily Living (BADL)	YES = 263 n (%)	NO = 580 n (%)	TOTAL = 843 N (%)
**Bathing**			
Dependent	7 (31.8)	15 (68.2)	22 (100.0)
Independent	256 (31.2)	565 (68.8)	821 (100.0)
**χ** ^**2**^ **= 0.004 df = 1; p = 0.949**			
**Dressing**			
Dependent	5 (33.3)	10 (66.7)	15 (100.0)
Independent	258 (31.2)	570 (68.8)	828 (100.0)
**χ** ^**2**^ **= 0.032 df = 1; p = 0.857**			
**Toileting**			
Dependent	8 (44.4)	10 (55.6)	18 (100.0)
Independent	255 (30.9)	570 (69.1)	825 (100.0)
**χ** ^**2**^ **= 1.504 df = 1; p = 0.220**			
**Transferring**			
Dependent	6 (24.0)	19 (76.0)	25 (100.0)
Independent	257 (31.4)	561 (68.6)	818 (100.0)
**χ** ^**2**^ **= 0.622 df = 1; p = 0.430**			
**Continence**			
Dependent	11 (40.7)	16 (59.3)	27 (100.0)
Independent	252 (30.9)	564 (69.1)	816 (100.0)
**χ** ^**2**^ **= 1.183 df = 1; p = 0.277**			
**Feeding**			
Dependent	2 (33.3)	4 (66.7)	6 (100.0)
Independent	261 (31.2)	576 (68.8)	837 (100.0)
**χ** ^**2**^ **= 0.013 df = 1; p = 0.910**			
**Overall BADL**			
Dysfunctional	15 (34.9)	28 (65.1)	43 (100.0)
Functional	248 (31.0)	552 (69.0)	800 (100.0)
**χ** ^**2**^ **= 0.287 df = 1; p = 0.592**			

Lifestyle habits of the respondents showed no significant association with reported snoring. However, higher proportion of respondents who engaged in some physical activities (31.3% vs 30.4%), drank alcohol (37.7% vs 30.8%) and coffee (35.5% vs 31.0%) reported snoring Table 3.

**Table 3:** Lifestyle habits by prevalence of snoring

	SNORING		
Lifestyle habits	YES = 263 n (%)	NO = 580 n (%)	TOTAL = 843 N (%)
**Alcohol intake**			
Yes	20 (37.7)	33 (62.3)	53 (100.0)
No	243 (30.8)	547 (69.2)	790 (100.0)
**χ** ^**2**^ **= 1.126 df = 1; p = 0.289**			
**Tobacco**			
Yes	5 (31.2)	11 (68.8)	16 (100.0)
No	258 (31.2)	569 (68.8)	827 (100.0)
**χ** ^**2**^ **= 0.000 df = 1; p = 0.964Ϯ**			
**Coffee**			
Yes	11 (35.5)	20 (64.5)	31 (100.0)
No	252 (31.0)	560 (69.0)	812 (100.0)
**χ** ^**2**^ **= 0.275 df = 1; p = 0.599**			
**Cannabis**			
Yes	2 (25.0)	6 (75.0)	8 (100.0)
No	261 (31.3)	574 (68.7)	835(100.0)
**χ** ^**2**^ **= 0.145 df = 3; p = 0.704 Ϯ**			
**Engagement in physical activities**			
Yes	221 (31.3)	484 (68.7)	705 (100.0)
No	42 (30.4)	96 (69.6)	138 (100.0)
**χ** ^**2**^ **= 0.045 df = 1; p = 0.832**			
**Level of physical activities ϮϮ (n =705)**			
Low level of activity	25 (34.2)	48 (65.8)	73 (100.0)
Moderately active	133 (29.1)	324 (70.9)	457 (100.0)
Very active	63 (36.0)	112 (64.0)	175 (100.0)
**χ** ^**2**^ **= 3.115 df = 2; p = 0.211**			

Ϯ Yates corrected

Ϯ Ϯ Respondents who engaged in physical activities

Physical characteristics of the respondents by prevalence of snoring are shown in Table 4. Among the male respondents, the prevalence of reported snoring was significantly associated with neck circumference (NC) >43 cm, body mass index (BMI) =30 kg/m^2^ and waist circumference (WC) =94 cm. Males respondents who snored had a higher waist-hip ratio (WHR) compared with men who did not snore (34.7% vs 21.7%) without a statistical difference. Among the females, the prevalence of reported snoring had statistical association with NC >40 cm, BMI =30 kg/m^2^ and WC =90 cm. In general, oropharyngeal crowding which was assessed using Mallampati score showed a very strong association with the prevalence of snoring and the degree of the oropharyngeal crowding.

**Table 4:** Physical characteristics by prevalence of snoring

	SNORING		
	YES = 263 n (%)	NO = 580 n (%)	TOTAL = 843 N (%)
**Waist-Hip Ratio (WHR)**			
**Male** < 1.00	10 (21.7)	36 (78.3)	46 (100.0)
≥ 1.00	102 (34.7)	192 (65.3)	294 (100.0)
**χ** ^**2**^ **= 3.022 df = 1; p = 0.082**			
**Female** < 0.85	21 (27.3)	56 (72.7)	77 (100.0)
≥ 0.85	130 (30.5)	296 (69.5)	426 (100.0)
**χ** ^**2**^ **= 0.372 df = 1; p = 0.568**			
**Neck Circumference (NC)**			
Male ≤ 43cm	92 (29.3)	222 (70.7)	314 (100.0)
> 43cm	20 (76.9)	6 (23.1)	26 (100.0)
**χ** ^**2**^ = 24.653 df = 1; p <0.0001*			
Female ≤ 40cm	141 (29.1)	343 (70.9)	484 (100.0)
> 40cm	10 (52.6)	9 (47.4)	19 (100.0)
**χ** ^**2**^ = 4.806 df = 1; p = 0.028*			
**Body Mass Index (BMI)**			
Male < 30kg/m^2^	89 (30.5)	203 (69.5)	292 (100.0)
≥ 30kg/m^2^	23 (47.9)	25 (52.1)	48 (100.0)
**χ** ^**2**^ = 5.674 df = 1; p = 0.017*			
Female < 30kg/m^2^	81 (24.1)	255 (75.9)	336 (100.0)
≥ 30kg/m^2^	70 (41.9)	97 (58.1)	167 (100.0)
**χ** ^**2**^ = 16.842 df = 1; p <0.0001*			
**Waist Circumference (WC)**			
Male < 94cm	50 (27.3)	133 (72.7)	183 (100.0)
≥ 94cm	62 (39.5)	95 (60.5)	157 (100.0)
**χ** ^**2**^ = 5.664 df = 1; p = 0.017*			
Female < 80cm	8 (17.4)	38 (82.6)	46 (100.0)
≥ 80cm	143 (31.3)	314 (68.7)	457 (100.0)
**χ** ^**2**^ = 3.844 df = 1; p = 0.050*			
**Oropharyngeal crowding (Mallampati classification)**			
**I**	4 (9.8)	37 (90.2)	41 (100.0)
**II**	55 (24.9)	166 (75.1)	221 (100.0)
**III**	141 (33.9)	275 (66.1)	416 (100.0)
**IV**	63 (38.2)	102 (61.8)	165 (100.0)
**χ** ^**2**^ = 18.041 df = 3; p <0.0001*			

* Significant at 5% level of significance

**Figure 2:**
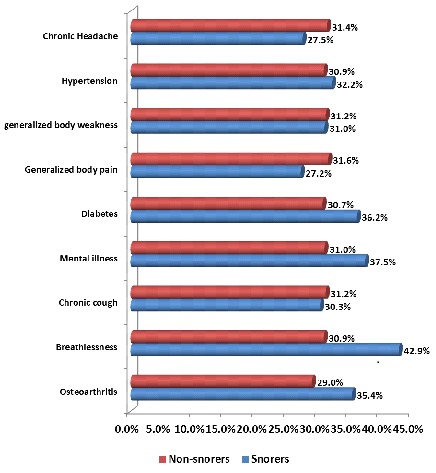
Prevalence of common morbidities in those with and without snoring

Figure 2 describes the prevalence of common morbidities in those with and without snoring. The prevalence of osteoarthritis (35.4% vs 29.0%), breathlessness (42.9% vs 30.9%), mental illnesses (37.5% vs 31.0%), diabetes mellitus (36.2% vs 30.7%) and hypertension (32.2% vs 30.9%) was higher among snorers than in those without snoring. However, no statistical association was found between these common morbidities and snoring.

Logistic regression was carried out on variables which showed significant association with reported snoring by sex. This is shown in Table 5. Among the elderly men, NC >43 cm was the most significant factor to snoring (OR = 6.005; 95% CI = 2.150 – 16.770). While among the elderly women, BMI =30 kg/m^2^ (OR = 2.028; 95% CI = 1.344 – 3.061) and moderate to severe oropharyngeal crowding (Mallampati classes III & IV) (OR = 1.639; 95% CI = 1.057 – 2.543) were the most significant factors to snoring.

**Table 5:** Logistic regression of significant factors associated with snoring

	β	Wald	p	OR	95.0% C.I. for OR	
					Lower	Upper
**MALES** Neck Circumference >43cm	1.793	11.702	0.001*	6.005	2.150	16.770
BMI ≥30kg/m^2^ Waist circumference ≥94cm	0.120 0.207	0.090 0.596	0.764 0.440	1.127 1.230	0.516 0.727	2.460 2.081
Mallampati Classes III&IV Constant	0.528 −2.655	0.285 17.260	0.064 0.000	1.696 0.070	0.969	2.967
**FEMALES** Neck Circumference >40cm	0.599	1.530	0.216	1.820	0.705	4.702
BMI ≥30kg/m^2^ Waist circumference ≥80cm	0.707 0.388	11.341 0.870	0.001* 0.351	2.028 1.474	1.344 0.652	3.061 3.331
Mallampati Classes III&IV Constant	0.494 −2.553	4.863 13.891	0.027* 0.000	1.639 0.078	1.057	2.543

* Significant at 5% level of significance

## Discussion

In this study, almost a third of the elderly reported snoring, with a quarter of the respondents also being habitual snorers. These findings were similar to the reports of previous studies on adult Nigerians by Sogebi et al and Olanisun et al as there was no Nigerian study which had specifically studied elderly Nigerians [1, 6]. However, the prevalence of snoring was higher among the elderly Turks (42%), Taiwanese (51.9%) and Germans (64.4%) compared with the elderly in our study [14, 15]. This difference may be due to cultural and methodological differences as it has been demonstrated that patients are not wholly accurate in their assessment of their own problems and therefore discrepancies may occur [16]. Similarly among the elderly, what constitutes a serious problem for one snorer or their partner, or both, may not necessarily be a problem for others [4]. Due to the fact there is still some uncertainty about the definition of snoring, we employed the most commonly used method for estimating the prevalence of snoring [6].

Almost all studies reported higher prevalence of snoring among males compared with the females across all age groups [1, 2, 6, 14]. Similarly in our study, more of the elderly men reported that they were snoring compared with the elderly women, though this difference was not statistically significant. None of the socio-demographic factors such as age, marital status and educational attainment showed any significant association with snoring among the respondents. However, the prevalence of snoring increased with the educational levels and was higher among those currently in marriage among the respondents. There was no consistency in the reports of studies concerning the association between the socio-demographic factors and snoring in the elderly [4, 17]. Therefore, more longitudinal population based studies focussing specifically on elderly are needed to elucidate the association between these socio-demographic factors and snoring in our setting.

There is a strong association between the measures of general obesity (BMI) and wide neck circumference (NC) with snoring. These associations were found independently in both sexes. However, the measure of central obesity (waist-hip ratio (WHR)) was not significantly associated with snoring. This as noted in previous studies was due to the reduction in pharyngeal airway diameter and resistance produced by deposits of adipose tissue in obese individuals [18–20]. Pharyngeal resistance correlates with increasing general obesity, with an obese individual having a 2.6-fold excess risk of becoming habitual snorers [6]. The association between wide NC (males >43 cm and females >40 cm) with snoring was not surprising, as NC has been found to be an important predispositions for obstructive sleep apnoea [5]. Multivariate analysis showed male respondent with a wide NC to be 6.0 times at risk of becoming a snorer. While, a female respondent with general obesity (BMI =30kg/m^2^) and moderate to severe oropharyngeal crowding (Mallampati III & IV) have a 2.0 and 1.6 excess risks of snoring respectively.

Though, snoring is the major symptom of sleep disordered breathing (SDB) but is not synonymous with SDB [14]. Snoring is characterized by sleepy patients with fragmented sleep rather than apnoea, the presence of snoring implies an elevated upper airway resistance with limited inspiratory flow [21]. Such episodes of flow limitation are frequently terminated by an arousal, so-called respiratory effort-related arousals (RERA), which lead to obstructive sleep apnoea (OSA) [14]. Complaints of tiredness after sleeping and on awakening were commoner among the female respondents. Similarly, very loud snoring was more prevalent among the female respondents. However, habitual snoring (snoring =3 times a week) was more common among male respondents.

In our study, higher proportions of respondents with osteoarthritis, breathlessness, mental illnesses (such as depression, anxiety and dementia), diabetes mellitus and hypertension were snorers when compared with those without these morbidities. Though, the prevalence of these common morbidities was not significantly associated with snoring. Studies have shown that snoring in individuals with osteoarthritis may be due to disruption in slow wave sleep leading to unrefreshed sleep, diffuse musculoskeletal pain, tenderness and fatigue [22]. The possible mechanism to developing diabetes mellitus among snorer was thought to be through insulin resistance via elevated sympathetic tone and intermittent hypoxia [23]. Studies have been inconsistent concerning the development of hypertension among snorers. While, some studies have confirmed that snoring is highly correlated with hypertension and other cardiovascular disease [24]. Lugaresi et al. determined that snoring is only associated with acutely elevated of blood pressure [25]. However, the Sleep Heart Health Study with other studies identified no significant association between self-reported snoring and hypertension or cardiovascular disease [14, 26, 27].

## Conclusion

The prevalence of reported snoring was high among elderly in our setting and preventable risk factors such as obesity, wide neck circumference and BMI which premise on lifestyle modification were the most significant factors found. Concerted efforts should be made by healthcare workers to address general and central obesity which have assumed a public health importance. Health education based on simple lifestyle measures such as regular exercise, dietary and weight controls should be inculcated into routine clinic practice.

**Limitations:** The potential limitation of this study is the failure to employ a gold standard diagnostic test, namely, an overnight polysomnography (PSG). We had based our diagnosis on reported snoring as sleep laboratories are few in the Nigeria and where available, are not affordable for elderly who depended mainly on pension which were not often forth-coming.
